# Integrative structural annotation of *de novo* RNA-Seq provides an accurate reference gene set of the enormous genome of the onion (*Allium cepa* L.)

**DOI:** 10.1093/dnares/dsu035

**Published:** 2014-10-31

**Authors:** Seungill Kim, Myung-Shin Kim, Yong-Min Kim, Seon-In Yeom, Kyeongchae Cheong, Ki-Tae Kim, Jongbum Jeon, Sunggil Kim, Do-Sun Kim, Seong-Han Sohn, Yong-Hwan Lee, Doil Choi

**Affiliations:** 1 Department of Plant Science, Seoul National University, Seoul, Korea; 2 Interdisciplinary Program in Agricultural Genomics, Seoul National University, Seoul, Korea; 3 Korea Bioinformation Center, Korea Research Institute of Bioscience and Biotechnology, Daejon, Korea; 4 Plant Genomics and Breeding Institute, Seoul National University, Seoul, Korea; 5 Department of Horticulture, Institute of Agriculture and Life Science, Gyeongsang National University, Jinju, Korea; 6 Department of Agricultural Biotechnology, Seoul National University, Seoul, Korea; 7 Department of Plant Biotechnology, Biotechnology Research Institute, Chonnam National University, Gwangju, Korea; 8 National Institute of Horticultural and Herbal Science, RDA, Suwon, Korea; 9 National Institute of Agricultural Science, RDA, Suwon, Korea

**Keywords:** *de novo* transcriptome, gene prediction, reference gene set, onion, non-coding sequence

## Abstract

The onion (*Allium cepa* L.) is one of the most widely cultivated and consumed vegetable crops in the world. Although a considerable amount of onion transcriptome data has been deposited into public databases, the sequences of the protein-coding genes are not accurate enough to be used, owing to non-coding sequences intermixed with the coding sequences. We generated a high-quality, annotated onion transcriptome from *de novo* sequence assembly and intensive structural annotation using the integrated structural gene annotation pipeline (ISGAP), which identified 54,165 protein-coding genes among 165,179 assembled transcripts totalling 203.0 Mb by eliminating the intron sequences. ISGAP performed reliable annotation, recognizing accurate gene structures based on reference proteins, and *ab initio* gene models of the assembled transcripts. Integrative functional annotation and gene-based SNP analysis revealed a whole biological repertoire of genes and transcriptomic variation in the onion. The method developed in this study provides a powerful tool for the construction of reference gene sets for organisms based solely on *de novo* transcriptome data. Furthermore, the reference genes and their variation described here for the onion represent essential tools for molecular breeding and gene cloning in *Allium* spp.

## 1. Introduction

The onion (*Allium cepa*) belongs to the family Amaryllidaceae, containing over 300 species; of which, 70 have been cultivated for 4,700 years or more.^1^ The onion is one of the major vegetable crops in the world. In 2012, onions were grown in 170 countries with global production of 87 million tons (http://faostat.fao.org/). Onions contain outstanding levels of polyphenols, vitamins, and sulphur-containing compounds, which are responsible for their pungency.^2–4^ Those compounds also affect various aspects of human health, including support for bone and connective tissues, anti-inflammatory effects, diabetes prevention, digestive tract health, and cancer protection.^2^ Hence, many traits were developed or are under development for onion breeding, including bulb shape, bulb colour, bulb size, flowering time, pungency, nutritional value, and disease resistance.

Despite the importance and significance of *Allium* species as major vegetable crops with nutritional and medicinal values, poor genomic information is available because of the enormous size of the genome (16.3 Gb).^5^ Although sequencing technologies have advanced rapidly in terms of higher throughput and longer read lengths, analysis of the complex and huge genome of the onion, a non-model plant, has remained a Herculean task.^6,7^ Currently, only a few information resources on the onion genome are available, including 511 proteins and 20,159 expressed sequence tags (ESTs) in GenBank, and those are insufficient for use in molecular breeding. Therefore, alternative sources of genomic information about the onion are required and have begun to be developed through transcriptome sequencing.^5^

High-throughput RNA sequencing (RNA-Seq) is a powerful and cost-effective tool to determine the structures of whole genes within genomes^8–13^ as well as to reveal a variety of biological information.^14–18^ Furthermore, for non-model organisms, it has been feasible to obtain whole transcripts without prior knowledge of a reference genome using *de novo* transcriptome analysis.^19–23^ Recent studies reported, however, that most RNA-Seq sequences and assembled transcripts contain various types of sequences such as introns, transposable elements (TEs), and non-coding RNAs, which are unnecessary information for construction of protein-coding genes.^16,24–26^ In most cases, gene annotation of *de novo* transcriptome assemblies is performed using ‘a six-frame translation’ approach without considering non-coding sequences.^17,27–33^ Large groups of non-coding sequences are therefore fully or partially included in the assembled transcripts, creating a potential barrier to accurate gene annotation. Constructing a precise and refined reference transcriptome is a prerequisite for reliability in further studies.

To this end, we generated a high-quality *de novo* assembly of onion transcriptome containing a large, annotated gene set using a standard gene-prediction pipeline by combining reference mapping and *ab initio* gene models. By validating the data using pre-existing gene sets, we have provided a reliable reference gene set for future genomic and genetic research on *Allium* spp. Furthermore, our study provides a useful model of a comprehensive approach to high-quality *de novo* transcriptome assembly and annotation in non-model species.

## 2. Materials and methods

### 2.1. Plant materials and cDNA library construction

A short-day type, doubled haploid onion with red bulbs (H6) and a lab-derived inbred line (SP3B) were used for cDNA synthesis and sequencing. Six weeks after planting vernalized bulbs, whole seedlings were harvested and then frozen in liquid nitrogen. Total RNA of each samples were extracted using TRIzol reagent (Invitrogen), and quality of extracted RNAs was inspected with Pico RNA chip and Pico6000 RNA reagent (Agilent Technologies) using a Bioanalyzer (Agilent Technologies). Strand-specific RNA-Seq paired-end libraries (insert size of 300–400 bp) were prepared according to the previous protocol,^34^ and the constructed libraries were used for transcriptome sequencing by Illumina HiSeq 2000.

### 2.2. *De novo* transcriptome assembly

The raw sequences of the onion transcriptome were processed using an in-house preprocessing pipeline to remove unnecessary sequences for the assembly.^35^ The preprocessing pipeline consisted of four steps. First, contaminating bacterial sequences were filtered out by mapping the reads to reference bacterial genomes in GenBank using Bowtie2 v2.0.0-beta7 (--local -D 15 -R 2 -N 0 -L 20 -i S,1,0.65).^36^ Secondly, duplicated short reads were removed. Thirdly, low-quality sequences with quality scores below Q20 were eliminated and sequences over 70 bp were remained using an in-house perl script. Finally, rRNA sequences were filtered out using SortMeRNA v1.9 (default parameter).^37^ The preprocessed sequences from H6 and SP3B and also a combined library of the sequences from both accessions were assembled using Velvet v1.2.08^38^ (-ins_length 400 -ins_length_sd 200) and Oases v0.2.06^39^ with a default parameter. To increase the assembly quality, iterative assemblies were performed to identify the *k*-mer values that gave the optimal total and average transcript lengths for each library. Thus, the optimal read lengths of 49, 47, and 53 bp were selected for the final assembly of H6, SP3B, and the combined library, respectively (Supplementary Fig. S1).

### 2.3. Structural and functional annotation

**Figure 1.**
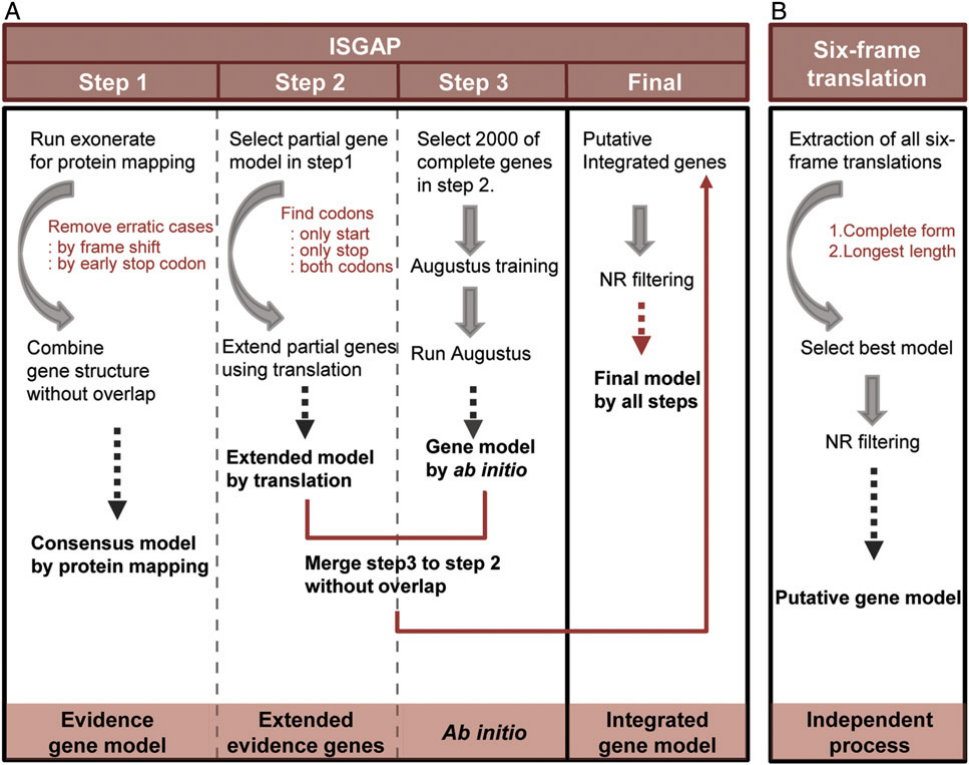
Integrated structural gene annotation pipeline (ISGAP). (A) ISGAP based on reference proteins and *ab initio* prediction. (B) The six-frame translation method as an independent process.

Structural gene annotation of the transcriptome was performed using the integrated structural gene annotation pipeline (ISGAP; Fig. 1). First, we used the reference gene annotations of monocot plants including *Musa acuminata* version 1.0,^40^ *Oryza sativa* RAP version 7.0,^41^ *Phyllostachys heterocycla* version 1.0,^42^ *Sorghum bicolor* version 1.0,^43^ and *Brachypodium distachyon* version 1.0^44^ to perform protein alignments using Exonerate v2.2.0^45^ with parameters --percent 30 and --maxintron 50,000 to find gene structures within the assembled transcripts. We then merged the detected gene structures which have same exon–exon junctions and constructed consensus sequences to use as initial gene models in the first step. During that process, we removed gene models that included frame shifts and early stop codons as well as remained one of the gene structures, which derived from a larger amount of evidence proteins and have a higher mapping score than other gene models in same regions. In the second step, partial genes in the initial gene models were extended through additional translation based on fixed frame of each gene model starting from five or three prime end of the partial genes to start or end region of the assembled transcript until translation of start or stop codon. In the third step, we constructed a training set of onion gene models using 2,000 of the complete genes generated by the second process, and we then ran Augustus^46^ using the training set. After filtering the abnormal gene models from Augustus, the initial gene models were integrated into a new gene model by extending the reference proteins without overlap. In the final step, after filtering the resulting gene models against the NR database in GenBank, we determined the final gene models.

For the final gene models, we extracted representative gene models remaining one of the genes, which have a longest complete form existing both start and stop codon in same locus of assembled transcripts. In case of no complete genes in the locus, we selected a longest partial gene as a representative gene model in the locus. The biological functions of the final gene models were assigned using InterProScan version 5-46,^47^ the plant proteins in the RefSeq database,^48^ and the Uniprot database,^49^ which contains the SWISS-PROT and TrEMBL databases with cut-off values *e*-value over 1*e*-5.

### 2.4. Evaluation of the predicted gene sets

The gene models predicted by ISGAP and the six-frame translation were validated by aligning the assembled transcripts to both the onion and the plant proteins of the RefSeq database^48^ in GenBank using Exonerate v2.2.0.^45^ To obtain accurate gene regions, we applied strict parameters for both validations (--percent 70 and over 90% mapping coverage). All of the correctly aligned regions that allowed redundancy, except for cases where a frame shift and early stop codon were detected, were compared as independent query sequences to the gene models predicted by ISGAP and six-frame translation. The gene models were evaluated based on their ability to represent query sequences considering the matched region, strand, and frame.

### 2.5. Sequence variation between the two accessions

A comparative transcriptomic analysis was performed to detect variation between H6 and SP3B. To detect variation, a reference-guided assembly was conducted by mapping the preprocessed raw SP3B sequence data to the H6 assembly via Bowtie2 v2.0.0-beta7^36^ (default parameter) Samtools v0.1.18^50^ and SNPeff v3.3h^51^ (-minC 5 -minQ 20) identified sequence variation and classified type of the variation by region of the assembled transcripts. To find more reliable variants, we extracted the sequence variants with their bi-directional 50-mer flanking sequences and confirmed that the sequence variants were detected in the flanking regions of the SP3B assembly using the in-house perl script.

## 3. Results

### 3.1. Sequencing, assembly, and repeat annotation

We obtained 15.0 and 11.6 Gb of whole-transcriptome sequences from H6 and SP3B by Illumina HiSeq 2000 using 101-bp paired-end reads (Supplementary Table S1). After preprocessing, a total of 10.1 and 7.9 Gb of sequences were remained for H6 and SP3B, respectively, and were used for *de novo* assembly (Supplementary Table S1). Using the combined library, a total of 203.0 Mb were assembled into 165,179 transcripts (mean length = 1,228.9 bp; N50 = 1,756 bp; Supplementary Table S2). The length distribution of the assembly showed that 76,699 transcripts (76.8% of the total) were longer than 1,000 bp (Supplementary Fig. S2). In addition, the assembly of the H6 and SP3B libraries separately produced 108,450 and 94,051 transcripts with an average length of 1,271.2 and 1,211.8 bp, respectively.

To validate the three sets of assembled transcripts, we conducted BLASTN using 20,159 onion ESTs downloaded from GenBank, 18,393 (91.2%) of which were detected among the assembled transcripts from the combined library with 98% sequence identity (Supplementary Fig. S3A). Moreover, most (83.4%) of the ESTs were represented among the assembled transcripts with at least 98% sequence identity and 80% coverage. Additionally, we confirmed that most of the ESTs were matched and covered by the assemblies of the H6 and SP3B libraries using the same parameters (Supplementary Fig. S3B and C).

Prior to structural gene prediction, we performed repeat masking using a constructed *de novo* repeat library made from the assembly of the combined library. A total of 38.9 Mb (19.2%) of the transcript sequences were determined to be TE-related repeats, including classified and unclassified repeats (Supplementary Table S3). A recent review reported that highly conserved and multicopy genes such as histone and tubulin can be recognized as repeat sequences during repeat masking.^8^ To avoid missing conserved protein-coding genes, we masked only the classified TEs including long interspersed nuclear elements, short interspersed nuclear elements, and long terminal repeats (LTRs) in the assembly of the combined library. In the assembly of the H6 and SP3B libraries, classified TEs were also masked to achieve optimal gene prediction for each assembly (Supplementary Table S3).

### 3.2. Structural annotation

Protein-coding genes in the transcriptome have mostly been predicted by classical methods such as six-frame translation of transcripts.^17,27–33^ That approach has been controversial, however, because of intermixed intron regions in the transcriptome. In order to predict more reliable gene sets, we established ISGAP to compare and integrate various gene-prediction approaches (Fig. 1). ISGAP comprises various gene-prediction approaches such as six-frame translation, evidence-based gene models based on protein mapping, *ab initio* gene prediction, and combined gene models.

To structurally annotate the genes in the onion transcriptome, protein alignment of the assembled transcripts was carried out to detect gene structures (Fig. 1A). To extend the gene models extracted from the protein alignments using reference genes, we conducted additional translation for partial genes, which have no start or stop positions (Fig. 1A). Thus, 42,435 gene models containing 26,598 complete genes were generated using reference proteins for further analysis (Supplementary Table S4). After the construction of the training set of Augustus,^46^ 51,092 gene models were predicted by Augustus and used to generate combined gene models with the gene models previously produced by Step 2 in ISGAP. Finally, 54,165 total genes and 20,447 representative protein-coding genes were annotated as a reference gene set of the onion after filtering against the NR database (Fig. 1A and Table 1). For the H6 and SP3B assemblies, we performed gene prediction using the same pipeline (Table 1). Additionally, to compare the gene models from ISGAP, six-frame translation was conducted as an independent step, and 65,645 genes were obtained (Fig. 1B and Table 2). The gene sets predicted by ISGAP showed a longer average length than those predicted by six-frame translation (Table 2 and Supplementary Table S4).

**Table 1.** Statistics of the annotated onion gene sets from ISGAP

	Combined	H6	SP3B
Whole			
Number of genes	54,165	38,004	35,750
Total length (Mb)	59.6	42.3	40.6
Average length (bp)	1,100.4	1,112.1	1,136.5
Representative			
Number of genes	20,447	18,034	17,101
Total length (Mb)	22.0	20.5	19.4
Average length (bp)	1,075.0	1,135.2	1,134.9

**Table 2.** Detailed statistics for all the annotated gene sets from six-frame translation and ISGAP using the combined library

	Six-frame translation	Step 1^a^	Step 2^b^	Step 3^c^	Steps 2 + 3^d^	Final
Number of genes	65,645	42,435	42,435	51,092	61,852	54,165
Number of genes containing multiple exons	N/A	9,481	9,481	10,207	13,516	11,496
Number of introns	N/A	11,015	11,015	12,436	16,600	13,543
Average length of exons (bp)	945.0	752.9	859.8	886.1	813.6	880.3
Average length of introns (bp)	N/A	298.9	298.9	344.7	310.6	307.7

a Gene model derived from reference proteins.

b Extended gene model through the translation of partial genes in Step 2.

c *Ab initio* predicted a gene model.

d Integrated gene model of Steps 2 and 3.

### 3.3. Validation and comparison of the gene models from ISGAP and six-frame translation

**Figure 2.**
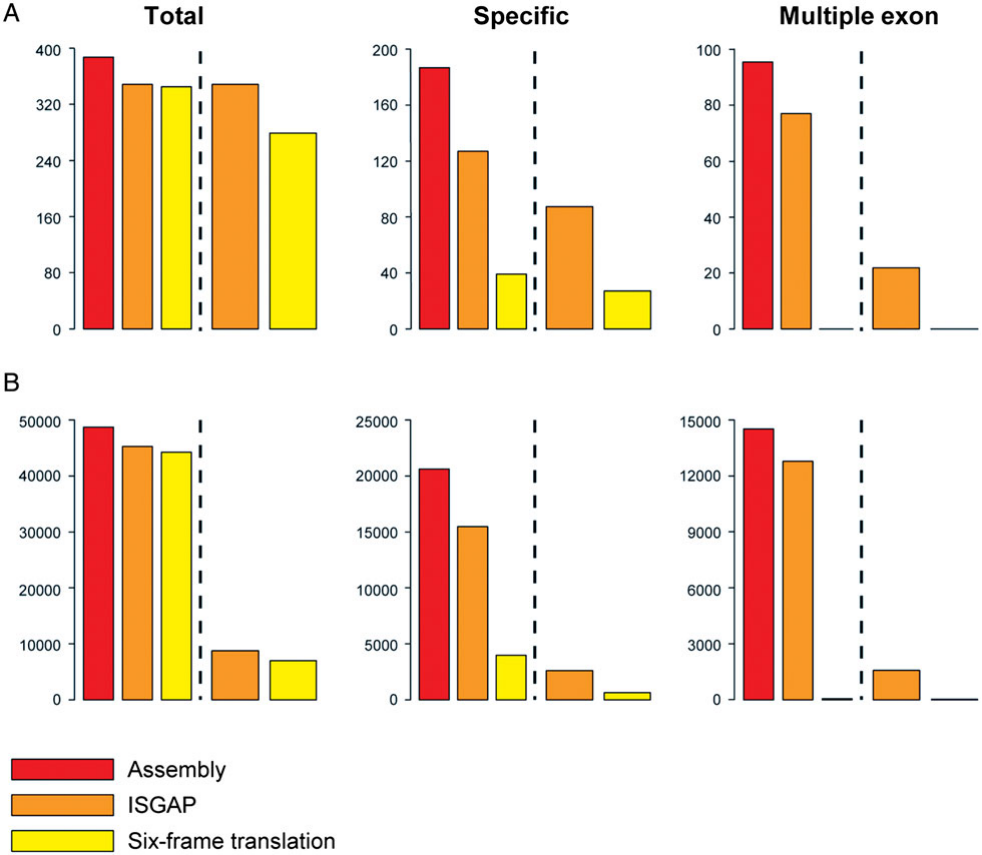
Comparison of the annotated onion gene sets predicted the combined library by six-frame translation and ISGAP. For the black dotted line, the left and right of the histogram represent the numbers of covered query sequences and predicted genes, respectively. (A) Validation of the predicted gene sets using 511 onion proteins. (B) Assessment of the predicted proteins against the plant proteins in the RefSeq database.

To evaluate the gene models predicted by ISGAP and six-frame translation, we aligned the onion proteins in GenBank to the assembled transcripts. With over 90% mapping coverage, 398 of 511 (77.9%) of the onion proteins were detected among 1,008 regions in the assembled transcripts (Fig. 2A and Supplementary Table S5). As a result, 348 and 281 genes predicted by ISGAP and six-frame translation, respectively, covered 351 and 344 (88.2 and 86.4%) of the mapped onion proteins with over 99% query coverage. Moreover, 866 and 746 (85.9 and 74.0%) of the mapped regions were represented by the genes predicted by ISGAP and six-frame translation, respectively (Supplementary Table S5).

Although the reference onion proteins matched more of the genes predicted by ISGAP than those predicted by six-frame translation, it was difficult to evaluate whole annotated genes because of the small number of known onion proteins in GenBank. To validate the whole genes in the annotated gene sets intensively, we aligned the assembled transcripts to the plant proteins in the RefSeq database.^48^ A total of 49,257 RefSeq proteins were discovered among 126,607 regions in the assembled transcripts (Fig. 2B and Supplementary Table S5). Similar numbers of proteins were detected with over 99% query coverage in both the ISGAP (45,724) and the six-frame translation (44,448) libraries. The numbers of mapped regions and the corresponding genes were different, however, between the ISGAP and six-frame translation libraries (Fig. 2B and Supplementary Table S5). The 8,324 genes predicted by ISGAP covered 114,593 (90.5%) of the mapped regions, whereas the 6,526 genes predicted by six-frame translation covered 93,256 (73.7%) of the mapped regions, suggesting that the number of precisely annotated genes in the ISGAP library was substantially higher than that in the six-frame translation library (Fig. 2).

To investigate differences between the gene sets, we selected and assessed all cases except both gene sets covered the same mapped regions in the validation results with 99% query coverage (Fig. 2 and Supplementary Table S5). For the onion reference proteins, 120 and 35 were represented by 94 and 27 gene annotations determined by ISGAP and six-frame translation, respectively (Fig. 2A). For the RefSeq proteins, 16,373 and 3,393 were represented by 2,341 and 543 gene annotations determined by ISGAP and six-frame translation, respectively (Fig. 2B). Although similar numbers of onion and RefSeq proteins were covered by genes from ISGAP and six-frame translation in the whole validation, the genes predicted by ISGAP in the extracted cases covered higher numbers of onion and RefSeq proteins, due to the fact that the same onion and RefSeq proteins were mapped in a different form on multiple transcripts (Supplementary Fig. S4A).

### 3.4. Assessment of multiple exon genes and annotated gene structures

Some of the onion and RefSeq proteins were mapped as genes containing multiple exons (Supplementary Fig. S4A). To validate those genes, including the multiple exons, we identified the cases where the onion and RefSeq proteins were mapped as genes containing multiple exons in both of the validation results (Fig. 2 and Supplementary Table S5). In the validation using the onion proteins, 92 proteins were mapped onto 124 of the mapped regions (Fig. 2A and Supplementary Table S5). For the proteins with over 99% query coverage, 23 genes predicted by ISGAP were represented by 78 (84.8%) of the onion proteins; however, the genes predicted by six-frame translation were not represented by any of the onion proteins (Fig. 2A). Moreover, 14,450 of the RefSeq proteins were detected among 24,719 of the mapped regions. With the same query coverage, 12,904 (89.3%) of the RefSeq proteins were correctly matched by 1,573 of the genes predicted by ISGAP (Fig. 2B and Supplementary Table S5). In contrast, only 6 (0.03%) of the RefSeq proteins were correctly matched by three of the genes predicted by six-frame translation (Fig. 2B and Supplementary Table S5). Rarely, the genes were fully matched to other RefSeq proteins mapped as single-exon genes (Supplementary Fig. S4B).

**Figure 3.**
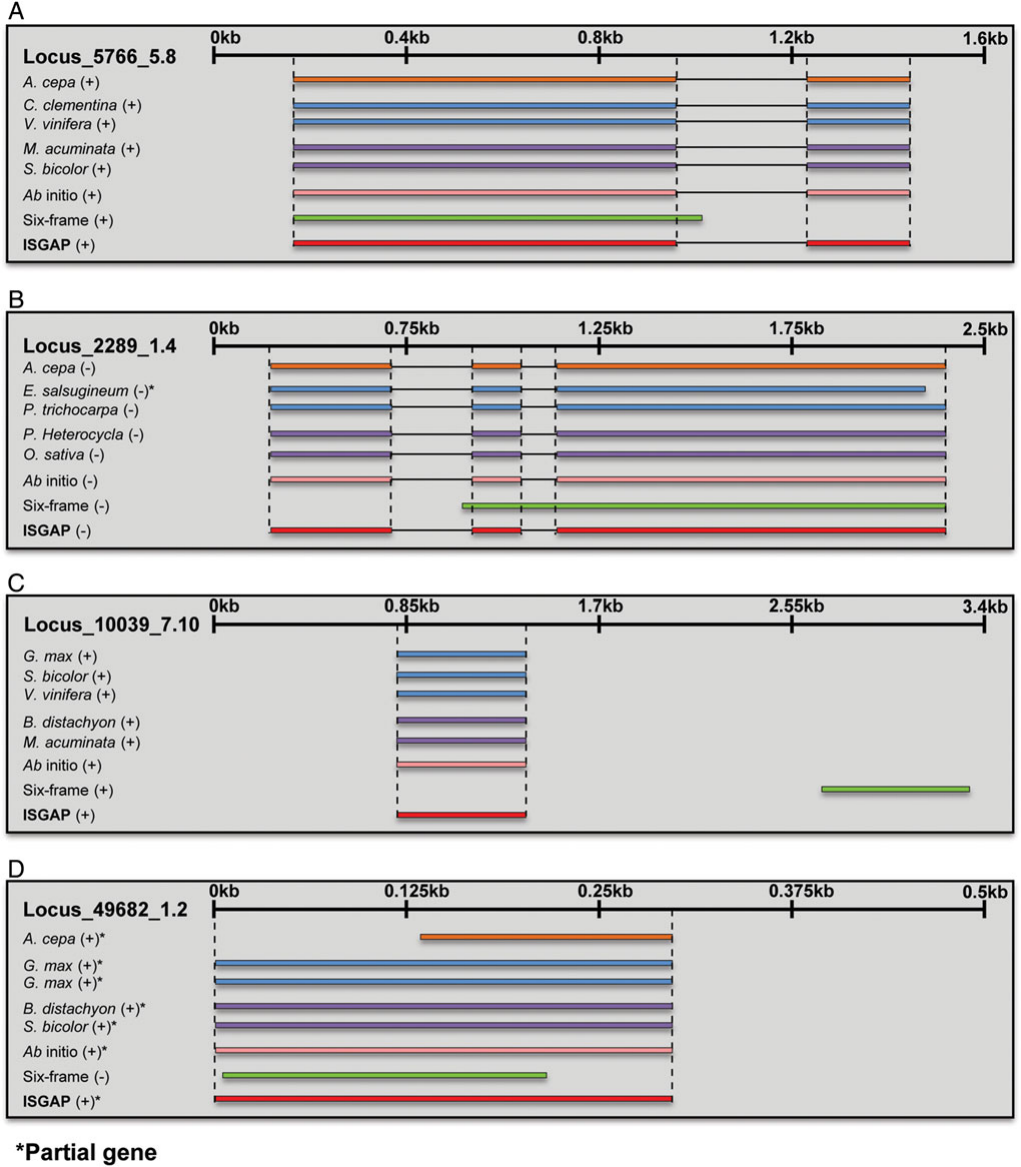
Representative cases of well-annotated genes predicted by ISGAP compared with the genes predicted by six-frame translation. The genes predicted by ISGAP and six-frame translation are shown, as well as the onion, RefSeq, reference, and *ab initio* gene models. The plus and minus signs in the brackets indicate the strand of mapped or predicted genes. (A and B) Cases of genes containing multiple exons; (C) gene annotation with the correct region. (D) Gene annotation with the correct strand.

Through the evaluation, we discovered miss-annotated genes derived from the six-frame translation. To verify the reasons for the miss-annotation, we examined the detailed structure of each gene model and identified representative cases among the validation results (Fig. 3). We found that the miss-annotations from the six-frame translation were caused by the retention of introns and the translation of the inappropriate region or strand (Fig. 3). For the corresponding regions, ISGAP successfully performed the gene annotations (Fig. 3). Hence, ISGAP could detect and extract accurate exon regions based on the structure of the reference protein and *ab initio* gene models, whereas six-frame translation could not.

### 3.5. Functional annotation and transcriptomic variation for the construction of genomic resources

**Figure 4.**
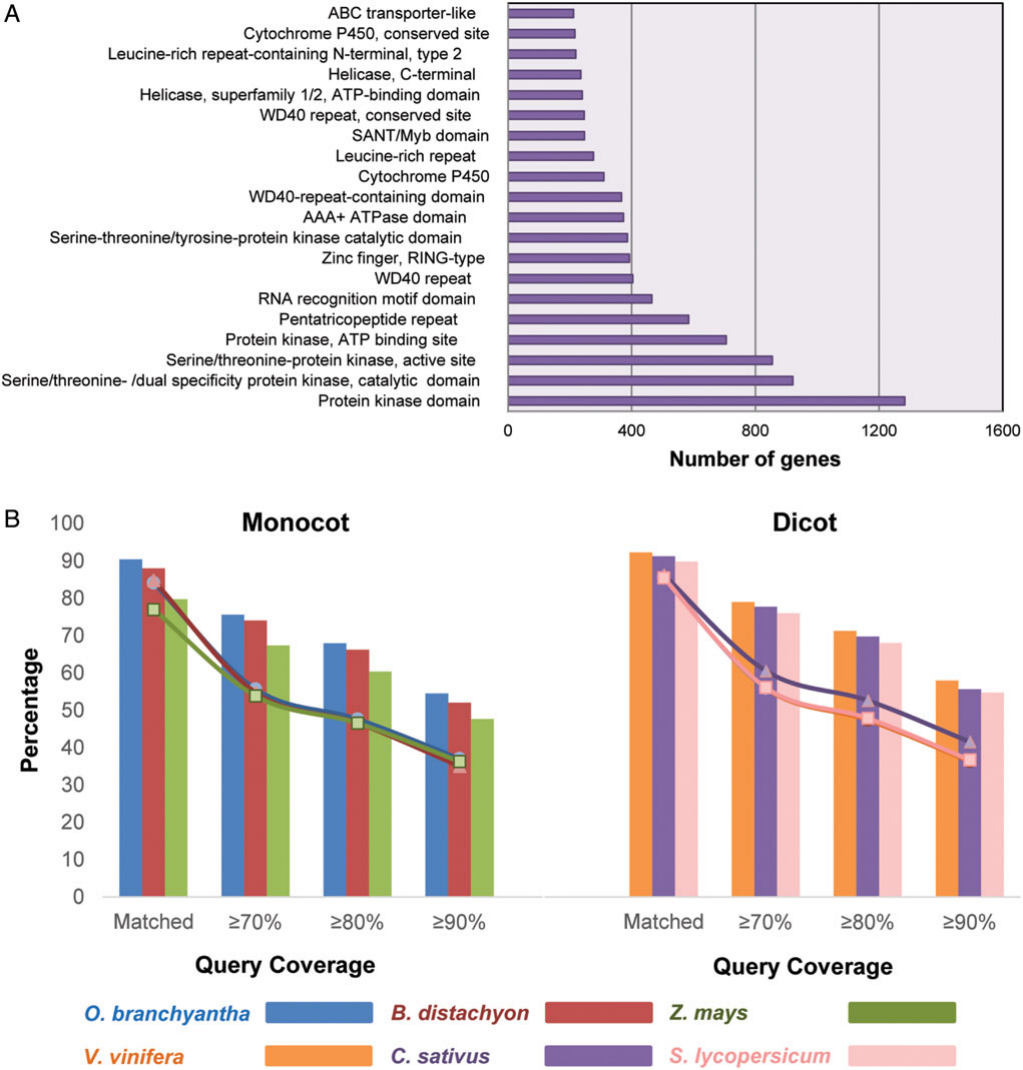
Distribution of biological functions and coverage graph for monocot and dicot plants. (A) Top 20 InterPro domains among the onion genes from the combined library. (B) Coverage graph of the onion genes in the assembly of the combined library on monocot and dicot plants. The line graph and histogram illustrate the proportions of onion genes and plant proteins in each species, respectively.

To date, the repertory of whole genes in the onion is unknown because of the absence of a reference genome. Therefore, we assigned functional information to the onion transcriptome based on the gene set predicted by ISGAP. We performed functional annotation using InterProScan version 5-46,^47^ containing Pfam, SMART, ProSite, and GO Term analysis. A total of 27,421 genes (50.6% of the total) were assigned functions based on the definitions of their domains (Supplementary Table S6). Among the annotated genes, the protein kinase domain was the most abundant and was detected in 1,283 genes (Fig. 4A). Moreover, the top 20 domains revealed the most actively expressed gene families in the onion (Fig. 4A).

To provide a more specific functional description of the onion genes, we conducted a BLAST analysis using the Uniprot database. A total of 44,710 onion genes (82.5% of the total) were discovered based on their best alignment with an *e*-value cut-off of 1*e*-5 (Supplementary Table S6). Moreover, using the RefSeq database, we performed additional functional annotations for the genes that were unassigned by the Uniprot database, and a total of 50,352 genes (93.0% of the total) were discovered in Uniprot or RefSeq databases and assigned biological functions (Supplementary Table S6). The same functional annotation was performed for the H6 and SP3B gene sets (Supplementary Table S7 and S8).

To estimate the coverage of the gene sets, we compared them to the proteins of each plant species in the RefSeq database via BLAST as an alternative approach to matching them with the whole genes of the onion. The predicted onion gene model covered from 60.5 to 68.0% of monocot proteins (*Oryza branchyantha*, *Brachypodium distachyon*, and *Zea mays*) and from 68.1 to 71.3% of dicot proteins (*Vitis vinifera*, *Cucumis sativus*, and *Solanum lycopersicum*) with cut-offs of 80% coverage and *e*-value of 1*e*-10 (Fig. 4B).

To build up the genomic resources for molecular marker development in the onion, we performed a comparative transcriptomic analysis to detect sequence variation between H6 and SP3B. We identified a total 50,064 SNPs and 14,016 InDels between H6 and SP3B (Table 3). For efficient marker development, we removed the SNPs and INDELs that did not have conserved flanking sequences in the assembly of SP3B. Consequently, a total 5,502 SNPs within protein-coding sequences and 5,942 SNPs within non-coding sequences were identified (Table 3 and Supplementary Table S9).

**Table 3.** Sequence variation between two cultivated onions, H6, and SP3B

	Whole variation				Confirmed variation^a^			
	Exon	Intron	Others^b^	Sum	Exon	Intron	Others^b^	Sum
SNPs	9,875	1,357	38,832	50,064	5,502	300	5,642	11,444
INDELs	766	834	12,416	14,016	47	19	431	497
Total	10,641	2,191	51,248	64,080	5,549	319	6,073	11,941

a Variations that have conserved flanking sequences in both assemblies.

b Regions except exon and intron.

## 4. Discussion

Since next-generation sequencing technology was developed, a large number of genomes have been sequenced from various organisms. Plants with very large genome sizes have not been fully sequenced because of features of the next-generation sequencing technology based on short reads. Because of the huge genome size of *Allium* spp., little genomic information has been deposited, creating an obstacle for the construction of an efficient molecular breeding platform. The emergence of high-throughput transcriptome sequencing offers an attractive solution for genomic studies of non-model organisms including the onion. Despite the advantages of transcriptome sequencing in terms of cost savings and reduced computing requirements, non-coding sequences in *de novo* transcriptomes have disrupted the construction of accurate genomic resources such as protein-coding gene libraries.^25^ Therefore, in-depth gene prediction for *de novo* transcriptome assembly is required to construct a fundamental reference gene set as an alternative genomic resource.

Typically, the six-frame translation approach has been used to extract protein-coding genes from *de novo* transcriptome assemblies. Six-frame translation is based, however, on the concept that assembled transcripts are composed of purified coding sequences, despite the presence of non-coding sequences. We generated *de novo* transcriptome assemblies with annotated gene sets of two onion accessions using ISGAP, an automated pipeline, with intensive validation. We focused our attention on constructing ISGAP to optimize accurate gene prediction for *de novo* transcriptome assembly with multiple gene-prediction steps (Fig. 1A). As the key steps of ISGAP, protein alignment was integrated to detect accurate exon regions, and an additional translation step was applied to extend the partial genes based on the detected exon regions (Fig. 1A). In addition, gene prediction using Augustus with a training step was integrated to obtain genes for transcript regions without matching reference proteins (Fig. 1A).

ISGAP predicted more genes accurately than six-frame translation did (Fig. 2), and it produced more specifically accurate reference genes (Fig. 2). The differences between the gene sets predicted by ISGAP and six-frame translation were mainly caused by the existence or non-existence of introns within the assembled transcripts (Supplementary Fig. 4A). Due to the retained intron sequences, the correct annotation by six-frame translation was interrupted, leading to miss-annotation or the construction of partial genes. For genes containing multiple exons, ISGAP could recognize the intron sequences and merge the exons by removing the introns, despite the protein-coding genes being broken into at least two exons separated by intron sequences in the assembled transcripts (Fig. 3A and B). Six-frame translation could not correctly annotate the genes containing multiple exons (Figs 2 and 3A and B). In addition, for the single-exon genes, six-frame translation often extracted miss-annotated genes by translation with an inappropriate region or strand (Fig. 3C and D). The protein-coding genes in the transcriptome need to be extracted by the consideration of the gene structure, and ISGAP successfully performed annotation based on reference proteins and *ab initio* gene models.

As shown in Fig. 4B, even if accurate comparison between the genes predicted by ISGAP and the plant proteins was difficult due to phylogenetic distance between the onion and the other plants, over 60% of the genes from each plant species were covered by half of the genes predicted by ISGAP with over 80% query coverage, suggesting that the gene set represented a large portion of the genes in the onion genome, although it was not enough to cover all of the genes in the onion. Nevertheless, transcriptome sequencing is still valuable for non-model organisms, owing to the absence of better alternative resources. The gene set predicted by ISGAP will contribute to the construction of a molecular breeding platform for the onion to boost the development of important traits such as shape, colour, size, pungency, and resistance to diseases. In conclusion, we expect that: (i) our comprehensive approach for accurate gene prediction using *de novo* transcriptome sequencing will provide a standard method for *de novo* transcriptome assembly in non-model organisms; (ii) the newly annotated gene set of the onion including various genomic resources such as SNPs and InDels will support molecular breeding and gene cloning in *Allium* spp.

## 5. Availability

The whole-transcriptome sequences of two cultivated onion are deposited in the GenBank database under SRP041918. The assembled transcripts of combined, H6, and SP3B libraries are deposited under the accession GBRQ01000000, GBRO01000000, and GBRN01000000, respectively. The version described in this paper is the first version. Further information, containing transcriptome assembly and annotated genes are uploaded on our website at http://onion.snu.ac.kr.

## Supplementary data

Supplementary data are available at www.dnaresearch.oxfordjournals.org.

## Funding

This work was supported by the Agricultural Genome Center of the Next-Generation Biogreen 21 Program (PJ008199012012), Rural Development Administration of the Korean government, and a grant (PJ710001-03) from Vegetable Breeding Research Center, an ARC program of Ministry for Food, Agriculture, Forestry and Fisheries, Republic of Korea. Funding to pay the Open Access publication charges for this article was provided by the Agricultural Genome Center of the Next-Generation Biogreen 21 Program (PJ008199012012), Rural Development Administration of the Korean government, and a grant (PJ710001-03) from Vegetable Breeding Research Center, an ARC program of Ministry for Food, Agriculture, Forestry and Fisheries, Republic of Korea.

## Supplementary Material

Supplementary Data
