# Supplementary material for: Integrative structural annotation of de novo RNA-Seq provides an accurate reference gene set of the enormous genome of the onion (Allium cepa L.)
Source: DNA Res. 2014 Oct 31;22(1):19–27. doi: 10.1093/dnares/dsu035 (PMC4379974; doi:10.1093/dnares/dsu035)
Supplement: Supplementary Data [file supp_22_1_19__index.html]

Integrative structural annotation of de novo RNA-Seq provides an accurate reference gene set of the enormous genome of the onion (Allium cepa L.) — Supplementary Data 

# Integrative structural annotation of *de novo* RNA-Seq provides an accurate reference gene set of the enormous genome of the onion (*Allium cepa* L.)

## Supplementary Data

Supplementary Data

**Files in this Data Supplement:**

- Supplementary Figure 1 - pdf file
- Supplementary Figure 2 - pdf file
- Supplementary Figure 3 - pdf file
- Supplementary Figure 4 - pdf file
- Supplementary Table 1 - pdf file
- Supplementary Table 2 - pdf file
- Supplementary Table 3 - pdf file
- Supplementary Table 4 - pdf file
- Supplementary Table 5 - pdf file
- Supplementary Table 6 - xls file
- Supplementary Table 7 - xls file
- Supplementary Table 8 - xls file
- Supplementary Table 9 - xls file
