# Supplementary material for: Integrative structural annotation of de novo RNA-Seq provides an accurate reference gene set of the enormous genome of the onion (Allium cepa L.)
Source: DNA Res. 2014 Oct 31;22(1):19–27. doi: 10.1093/dnares/dsu035 (PMC4379974; doi:10.1093/dnares/dsu035)
Supplement: Supplementary Data [file supp_dsu035_dsu035supp_figure1.pdf]

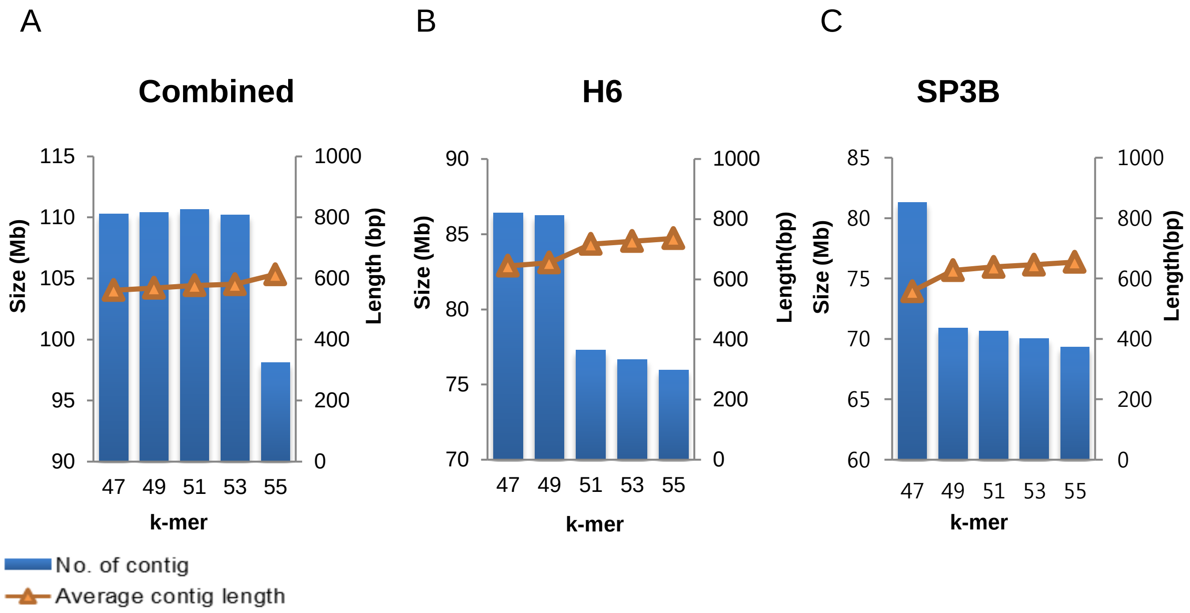

Figure S1. **Comparison of *de novo* assembly using various k-mer values.** Left and right of y-axis indicates total and average length of assembly respectively. The x-axis represents used k-mer values for assembly.
