# Supplementary material for: Integrative structural annotation of de novo RNA-Seq provides an accurate reference gene set of the enormous genome of the onion (Allium cepa L.)
Source: DNA Res. 2014 Oct 31;22(1):19–27. doi: 10.1093/dnares/dsu035 (PMC4379974; doi:10.1093/dnares/dsu035)
Supplement: Supplementary Data [file supp_dsu035_dsu035supp_figure3.pdf]

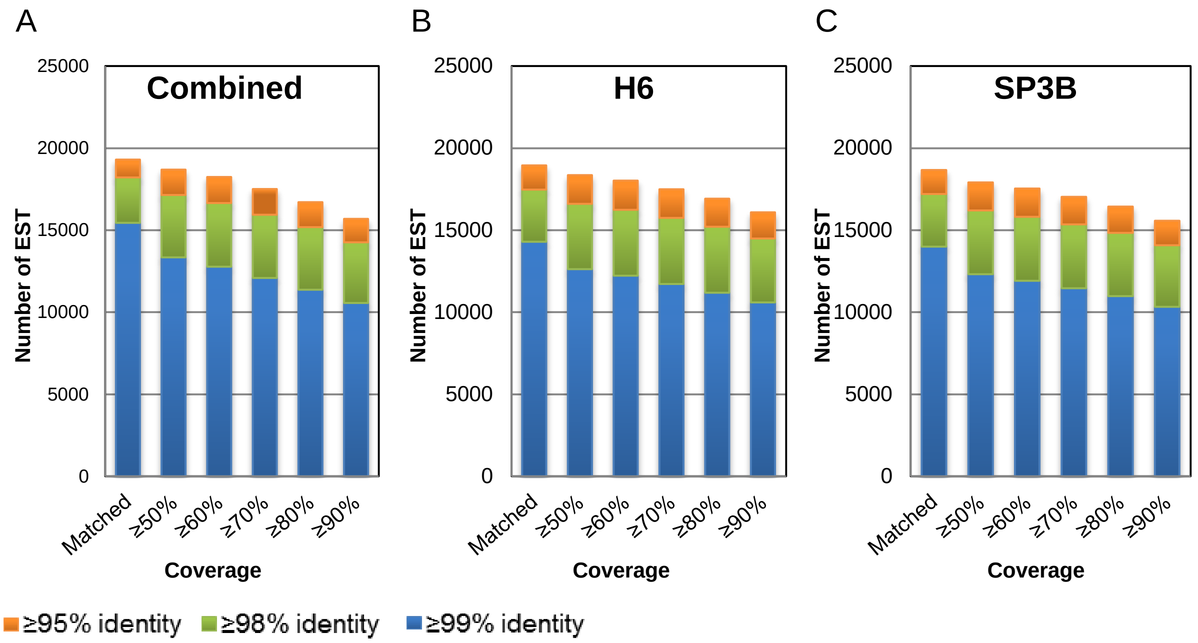

Figure S3. Validation of *de novo* assembly using 20,159 of onion ESTs. The x-axis indicates coverage of assembly for ESTs. The y-axis represents number of ESTs.
