# Supplementary material for: Integrative structural annotation of de novo RNA-Seq provides an accurate reference gene set of the enormous genome of the onion (Allium cepa L.)
Source: DNA Res. 2014 Oct 31;22(1):19–27. doi: 10.1093/dnares/dsu035 (PMC4379974; doi:10.1093/dnares/dsu035)
Supplement: Supplementary Data [file supp_dsu035_dsu035supp_figure4.pdf]

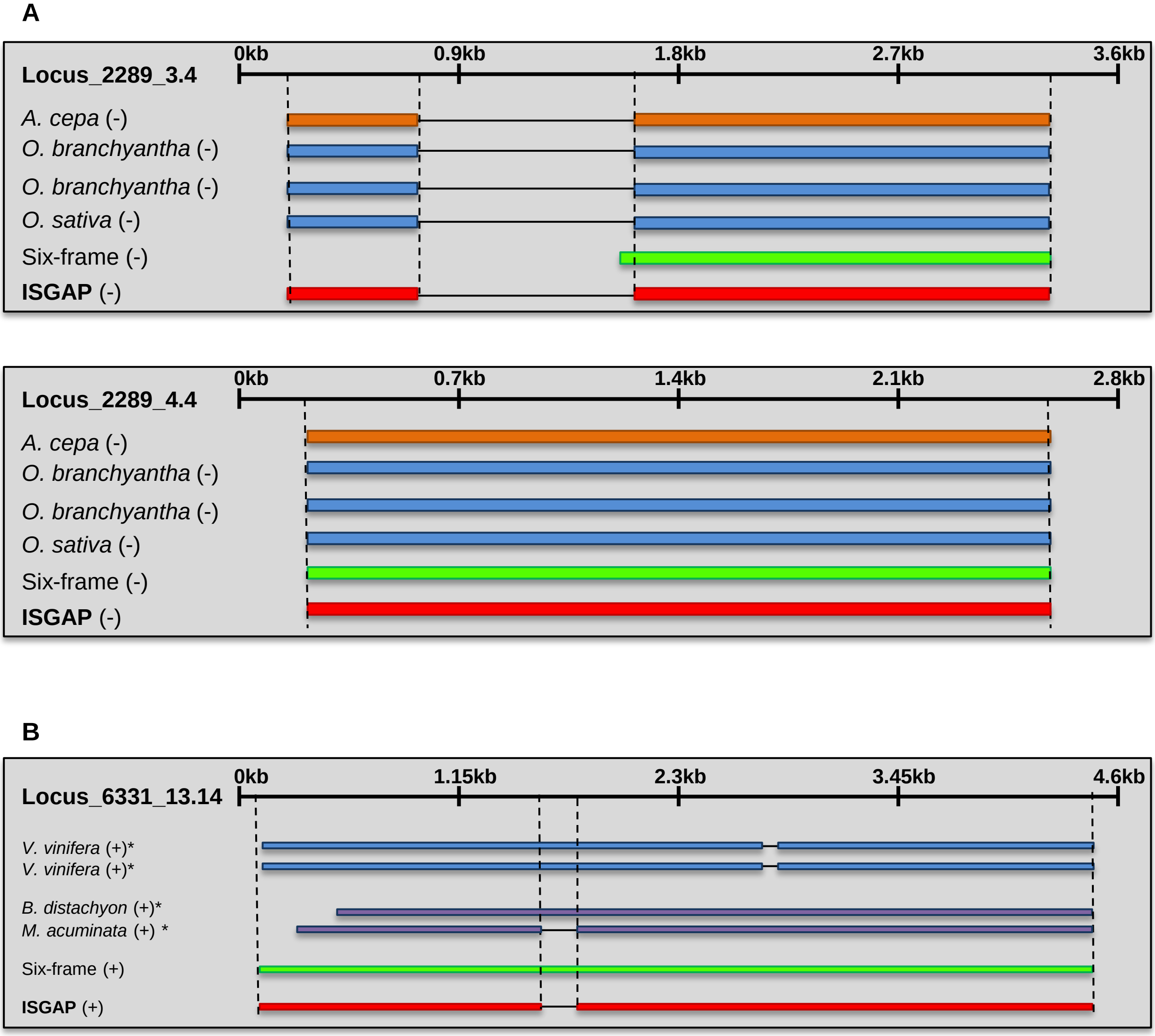

***Partial gene**

Figure S4. **Differential mapped structures of public proteins in multiple or single transcript.** Onion (orange bar), RefSeq plant proteins (blue bar) and evidence gene models (purple bar) were represented with gene models from ISGAP (red bar) and six-frame translated gene models (green bar). **(A)** The mapped structure of same onion and RefSeq proteins in differential transcripts. **(B)** The differentially mapped proteins in single transcript.
