# Supplementary material for: Integrative structural annotation of de novo RNA-Seq provides an accurate reference gene set of the enormous genome of the onion (Allium cepa L.)
Source: DNA Res. 2014 Oct 31;22(1):19–27. doi: 10.1093/dnares/dsu035 (PMC4379974; doi:10.1093/dnares/dsu035)
Supplement: Supplementary Data [file supp_dsu035_dsu035supp_table1.pdf]

Table S1. Statistics of preprocessed raw reads.

Cultivar	Raw data(Gb)	Step1[a](Gb)	Step2[b](Gb)	Step3[c](Gb)	Step[d](Gb)
**H6**	14.98	14.96	12.02	10.12	10.08
**SP3B**	11.57	11.57	9.55	7.94	7.91
**Total**	26.55	26.53	21.57	18.06	17.99

[a]Removal of bacterial genome using reference mapping
[b]Removal of duplicated reads
[c]Quality trimming (cut off = Q20)
[d]Removal of rRNA
