# Supplementary material for: Integrative structural annotation of de novo RNA-Seq provides an accurate reference gene set of the enormous genome of the onion (Allium cepa L.)
Source: DNA Res. 2014 Oct 31;22(1):19–27. doi: 10.1093/dnares/dsu035 (PMC4379974; doi:10.1093/dnares/dsu035)
Supplement: Supplementary Data [file supp_dsu035_dsu035supp_table2.pdf]

Table S2. Statistics of *de novo* transcriptome assembly.

Assembly	Total number	Base-pairs(Mbp)	N50(bp)	Mean(bp)	Max(bp)	Min(bp)
**H6**	108,450	137.9	1,790	1,271.2	16,429	200
**SP3B**	94,051	114.0	1,695	1,211.8	16,482	200
**Combined**	165,179	203.0	1,756	1,228.9	16,504	200
