# Supplementary material for: Integrative structural annotation of de novo RNA-Seq provides an accurate reference gene set of the enormous genome of the onion (Allium cepa L.)
Source: DNA Res. 2014 Oct 31;22(1):19–27. doi: 10.1093/dnares/dsu035 (PMC4379974; doi:10.1093/dnares/dsu035)
Supplement: Supplementary Data [file supp_dsu035_dsu035supp_table3.pdf]

Table S3. The amount of transposable elements (TEs) in onion transcriptome assembly.

Type of TEs	Combined		H6		SP3B	
	Length (bp)	% in assembly	Length (bp)	% in Assembly	Length (bp)	% in assembly
**SINEs**	181,919	0.09	128,334	0.09	90,903	0.08
**LINEs**	5,245,049	2.58	3,443,494	2.50	2,471,579	2.17
**LTR elements**	6,124,193	3.02	4,212,620	3.06	2,625,618	2.30
**DNA elements**	4,120,452	2.03	2,636,857	1.91	1,769,302	1.55
**Simple repeats**	1,708,443	0.84	1,142,352	0.83	918,098	0.81
**Low complexity**	412,511	0.20	268,572	0.19	232,315	0.20
**Unclassified**	21,090,264	10.39	14,403,114	10.45	11,097,869	9.74
**Total**	38,882,831	19.15	26,235,343	19.03	19,205,684	16.85
