# Supplementary material for: Integrative structural annotation of de novo RNA-Seq provides an accurate reference gene set of the enormous genome of the onion (Allium cepa L.)
Source: DNA Res. 2014 Oct 31;22(1):19–27. doi: 10.1093/dnares/dsu035 (PMC4379974; doi:10.1093/dnares/dsu035)
Supplement: Supplementary Data [file supp_dsu035_dsu035supp_table4.pdf]

Table S4. Summary of all annotated gene sets of combined library from each step of in-house pipeline.

	Independent process[a]	Step 1[b]	Step 2[c]	Step 3[d]	Step2+3[e]	Final[f]
**Number of genes**	65,645	42,435	42,435	51,092	61,852	54,165
**Number of complete genes**	53,071	3,704	26,598	33,154	35,995	32,791
**Number of partial genes**	12,574	38,731	15,837	17,938	25,857	21,374
**Total gene length (Mbp)**	62.03	40.26	45.96	56.28	63.83	59.60
**Avg length of gene (bp)**	945.00	948.43	1,082.98	1,101.83	1,031.96	1,100.40

[a]Gene model extracted by six-frame translation
[b]Gene model derived from evidence proteins
[c]Extended gene model through translation for partial genes in step 2
[d]*Ab initio* predicted gene model
[e]Integrated gene model of step 2 and 3
[f]Final gene set
