# Supplementary material for: Integrative structural annotation of de novo RNA-Seq provides an accurate reference gene set of the enormous genome of the onion (Allium cepa L.)
Source: DNA Res. 2014 Oct 31;22(1):19–27. doi: 10.1093/dnares/dsu035 (PMC4379974; doi:10.1093/dnares/dsu035)
Supplement: Supplementary Data [file supp_dsu035_dsu035supp_table5.pdf]

Table S5. Number of validated proteins and their corresponding genes from ISGAP and six-frame translation.

Data	Type	Mapped in Assembly		ISGAP			Six-frame translation		
		#Mapped Protein	#Mapped Region	#Mapped Protein	#Mapped Region	#Annotated Gene	#Mapped Protein	#Mapped Region	#Annotated Gene
Onion	Whole[a]	398	1,008	351	866	348	344	746	281
	Both[b]	322	693	322	693	255	322	693	255
	Specific[c]	188	315	120	173	94	35	53	27
	Single Exon[d]	396	884	351	767	326	344	746	281
	Multiple Exon[e]	92	124	78	99	23	0	0	0
RefSeq	Whole[a]	49,257	126,607	45,724	114,593	8,324	44,448	93,256	6,526
	Both[b]	42,126	87,168	42,126	87,168	5,984	42,126	87,168	5,984
	Specific[c]	21,056	39,439	16,373	27,425	2,341	3,393	6,088	543
	Single Exon[d]	47,047	101,888	43,970	93,595	6,752	44,443	93,251	6,524
	Multiple Exon[e]	14,450	24,719	12,904	20,998	1,573	6	5	3

[a]Whole cases in the validation
[b]Cases which both gene sets covered
[c]Cases that genes of ISGAP or six-frame translation specifically covered
[d]Cases that public proteins were mapped as single exon gene
[e]Cases for multiple exon containing genes
